# Emerging Cellular and Molecular Strategies for Enhancing Central Nervous System (CNS) Remyelination

**DOI:** 10.3390/brainsci8060111

**Published:** 2018-06-15

**Authors:** Mohammad Abu-Rub, Robert H. Miller

**Affiliations:** 1 Department of Neurology, George Washington University School of Medicine and Health Sciences, Washington, DC 20037, USA; aburub@gwu.edu; 2 Department of Anatomy and Regenerative Biology, George Washington University School of Medicine and Health Sciences, Washington, DC 20037, USA

**Keywords:** demyelination, remyelination, multiple sclerosis, OPCs, oligodendrocytes, MSCs

## Abstract

Myelination is critical for the normal functioning of the central nervous system (CNS) in vertebrates. Conditions in which the development of myelin is perturbed result in severely compromised individuals often with shorter lifespans, while loss of myelin in the adult results in a variety of functional deficits. Although some form of spontaneous remyelination often takes place, the repair process as a whole often fails. Several lines of evidence suggest it is feasible to develop strategies that enhance the capacity of the CNS to undergo remyelination and potentially reverse functional deficits. Such strategies include cellular therapies using either neural or mesenchymal stem cells as well as molecular regulators of oligodendrocyte development and differentiation. Given the prevalence of demyelinating diseases and their effects on the quality of life for affected individuals it is imperative that effective therapies are developed. Here we discuss some of the new approaches to CNS myelin repair that hold promise for reducing the burden of diseases characterized by myelin loss.

## 1. Introduction

There are many forms of demyelinating diseases in humans, and they are often typified by the loss or dysfunction of oligodendrocytes [1]. Although myelin loss per se is associated with conduction blocks along myelinated axons that often lead to clinical deficits, it is usually followed by some degree of axonal dysfunction or loss associated with the accrual of functional disability [2]. Remyelination is a regenerative process whereby myelin sheaths are restored, and although spontaneous remyelination is a normal physiological response after most demyelinating conditions [3,4], there is evident heterogeneity in the extent of remyelination, with an estimated 20–30% of people with multiple sclerosis (MS) showing extensive remyelination [5]. The study of developmental myelination, pathologic demyelination, and remyelination has been instrumental in setting the stage for this emerging field and is evidenced by the amount of work being undertaken to develop remyelinating therapies. Despite having achieved milestones at developing disease modifying therapies that halt the progression of diseases like MS, there is a paucity of directed therapies to repair or regenerate myelin [6]. Therefore, there is a need to understand mechanisms of remyelination and causes of remyelination failure to be able to better design strategies for enhancing remyelination. Here, we discuss some of these potential strategies, particularly in relation to myelin biology in health and disease.

## 2. What Is the Role of Myelin in the CNS?

**Figure 1:**
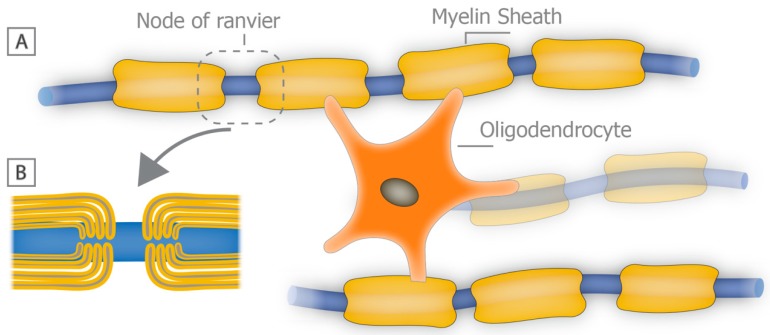
Structure of the myelin sheath in the central nervous system (CNS). (**A**) schematic diagram depicting a mature oligodendrocyte contributing to the myelin sheaths of three individual axons. Myelin sheaths are separated by bare segments of the axon, called nodes of Ranvier. Internodal myelin is shown in more detail in panel (**B**) where stacks of myelin are seen surrounding a segment of the axon.

The functioning of the central nervous system (CNS) relies heavily on the precise temporal and spatial connectivity of populations of neurons and their axonal processes. Central to this connectivity is the ability of axons to rapidly and effectively convey action potentials to their postsynaptic targets, which in most instances is dependent on their level of myelination. Myelin is composed of wraps or stacks of modified plasma membrane that act to insulate and protect segments of the axons all the while providing metabolic trophic support [7,8] (Figure 1). A primary function of myelin sheaths is to enhance the speed at which electrical impulses travel along axons and reduce the threshold required for action potential propagation. This is reflected in the discontinuous nature of myelin, where there is a high concentration of sodium ion channels at the intervening nodes of Ranvier [9]. Not all axons in the CNS are myelinated, but those that are myelinated tend to be long projection axons or in some cases inhibitory interneurons, where rapid flow of information is important and if such axons lose their myelin sheaths, their ability to effectively conduct information is compromised. This slowing or even failure of axonal conduction can in turn result in disturbances of motor and sensory function as seen clearly in demyelinating neurodegenerative diseases such as MS. Short term loss of myelin often leads to axonal conduction blocks and temporary axonal dysfunction that is likely to recover with remyelination [10]; however, long-term or chronic myelin loss without rapid and effective remyelination can lead to axonal degeneration with ensuing persistent functional deficits [11].

## 3. Myelin Biology: From Development to Regeneration

**Figure 2:**
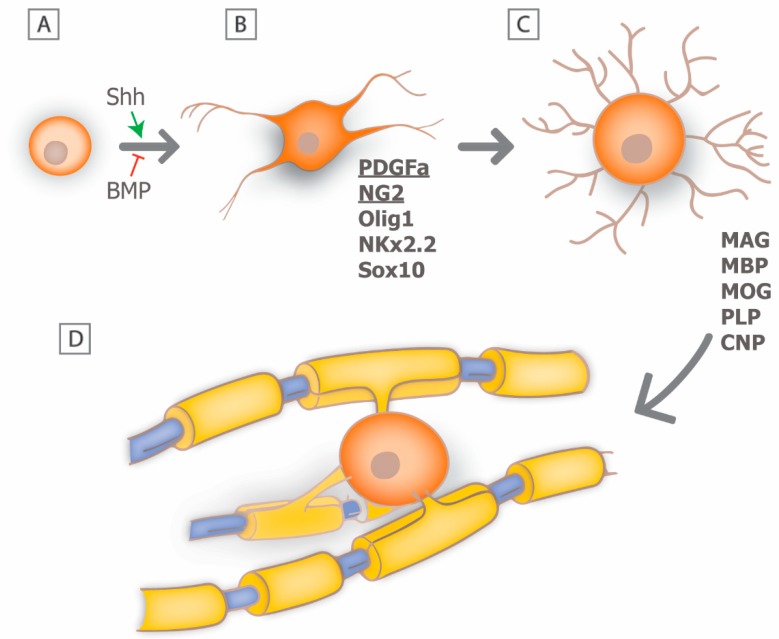
Schematic of the major stages of development of oligodendrocytes. (**A**) Neural stem cells are induced by Shh to produce (**B**) oligodendrocyte precursor cell (OPCs), which are uniquely identified by PDGFa and NG2, among others. Differentiation of OPCs into myelinating oligodendrocytes (**C**,**D**) is associated with an increase in cellular and molecular complexity including the expression of various myelin-specific proteins and lipids.

In the CNS, myelin is produced by post-mitotic oligodendrocytes and a single oligodendrocyte may myelinate multiple axons. Myelin regeneration following oligodendrocyte loss is dependent on the effective replacement of damaged cells, and much of the current understanding of oligodendrogensis comes from developmental studies [12,13]. In the brain and spinal cord, oligodendrocytes develop from a distinct population of multipotent precursor cells (oligodendrocyte precursors; OPCs) [14]. These cells are generated from neural stem cells and arise in distinct locations of the neural tube during late embryonic development, such as the ventral ventricular zone of the spinal cord, in response to distinct molecular cues. The molecular mechanism that mediates the early development of OPCs is now relatively well understood and involves the initial induction of OPC fate by neural patterning cues such as sonic hedgehog (Shh), and antagonism by members of the bone morphogenetic protein family (BMP) (Figure 2A) [15,16]. Subsequently, OPCs express a series of transcription factors including *Olig1*, *Nkx2.2*, *Sox10* and others combined with the repression of negative regulators of oligodendrocytic fate, such as Sox6 and Hes5 [17,18].

At the early stages of oligodendrocyte lineage commitment, OPCs express a characteristic molecular phenotype that includes expression of the platelet derived growth factor receptor alpha (PDGFRa) and the cell surface glycoprotein NG2 that allows for their unambiguous identification [19,20] (Figure 2B). OPCs are highly proliferative in response to growth factors such as PDGFa and fibroblast growth factor (FGF) and disperse widely throughout the developing CNS. Their migration is guided by dispersal cues such as Netrin 1 and an association with the developing vasculature [21]. Recent studies suggest that vascular associated migration is mediated by signaling through the Wnt pathway, which provides polarity and an effective substrate for cell translocation [22]. The differentiation of OPCs into premyelinating oligodendrocytes is associated with an increase in cellular complexity, an inhibition of proliferation, and the expression of myelin associated proteins and lipids, including myelin basic protein (MBP), myelin associated glycoprotein (MOG), proteolipid protein (PLP), and cyclic nucleotide phosphodiesterase (CNP) [23]. The transition from OPC to oligodendrocyte is also a period during which the cells are highly susceptible to the induction of cell death, and it has been proposed that in white matter tracts such as the optic nerve as many as 50% of the premyelinating oligodendrocytes undergo apoptotic cell death [24]. If premyelinating oligodendrocytes each associate with an appropriate population of axons they will mature into myelinating oligodendrocytes (Figure 2C) [25].

Each individual oligodendrocyte extends multiple processes that associate with an individual axon and myelinate a defined segment or internode (Figure 2D). The ability to image the formation of individual myelin sheaths in model systems such as zebrafish have provided critical insights into the mechanism of myelin sheath formation and growth [26,27,28,29,30,31]. For example, following initial axo-glial contact, one oligodendrocyte will extend multiple processes that contact multiple axons within a clearly defined time-window. Some of those processes are rapidly retracted suggesting that some form of axon selection process takes place. Interestingly, there is only a brief period during which myelination can take place, and oligodendrocytes are deemed incapable of extending additional internodes once they mature [30,32]. Those processes that are sustained, generate initial myelin segment domains that may either grow, remain constant, or shrink. It is now known that many of these changes are the result of activity-dependent secretion by axons, whereby inhibition of synaptic vesicle exocytosis in zebrafish resulted in shorter, thinner myelin sheaths [31]. Furthermore, control of the myelin segment growth is at the level of individual sheaths rather than at the level of oligodendrocyte cell body and is dependent on local calcium signaling [27,28,29]. It seems likely that electrical activity within the axon is important in regulating Ca^2+^ fluxes and modulating the extent of myelination [33,34,35]. For example, blocking electrical activity of retinal ganglion cells through preventing eye opening or following treatment with the Na^+^ channel blocker tetrodotoxin (TTX) significantly perturbs myelination of the optic nerve [33]. Similarly, following toxic demyelination with ethidium bromide, glutamatergic transmission between axons and OPCs directly modulate remyelination, such that when neuronal activity is blocked or synaptic transmission inhibited, OPCs do not differentiate into myelinating oligodendrocytes, but rather remain in a proliferative state [36]. This phenomenon could be responsible for failure of remyelination in chronic MS lesions.

Recent evidence suggests that myelin production persists throughout life, including changes in existing internodes, or even generation of new internodes by newly generated oligodendrocytes [37,38]. This form of myelin plasticity could have a direct effect on neuronal plasticity and in turn be critical for learning and memory. How the wrapping of the myelin sheath actually occurs is not well understood at a cellular level. Several lines of evidence suggest, however, that both biophysical properties of the myelin sheath proteins MBP and PLP contribute to selective membrane fusion while changes to the oligodendrocyte cytoskeleton, particularly the actin network, are essential to facilitate formation of the compact myelin sheath [39,40,41,42].

## 4. Mechanisms of Remyelination: Can We Recapitulate Developmental Signals?

A major challenge is to understand how our knowledge of developmental myelination can be utilized to promote myelin repair in the adult CNS. The so-called recapitulation hypothesis has emerged with the notion that developmental myelination and remyelination, should at least in theory, have similar processes, with regards to OPC expansion, migration, proliferation, and differentiation into myelinating oligodendrocytes. Although much of the current literature remains to this date biased towards development, there are some key findings in support of this hypothesis. For example, the rate limiting steps in generating new myelin may be at the level of OPC generation, differentiation or maturation. It is known that the many of the remyelinating oligodendrocytes come from local OPCs [43,44]. However, recent evidence suggests that neural precursor cells are a major source of myelinating oligodendrocytes which in some regions such as the rostral corpus callosum, outnumber those generated from OPCs [45]. In addition, many of the genes upregulated in OPCs following injury to the white matter resemble those associated with oligodendrocytes during development, such as *Olig2* and *Nkx2.2* [46]. One potential complication to using development as a guide to myelin repair is that the environment of the damaged CNS is significantly different from that in development and some critical signaling pathways, or the sequence of their expression, may be altered.

## 5. Insults with Associated Myelin Loss

There are a wide range of conditions in which the development or maintenance of myelin is compromised, either as a result of direct damage to the myelin sheath, or indirectly as a result of oligodendrocyte dysfunction or loss (reviewed in [47]). Whatever the underlying cause, myelin loss is generally associated with significant functional deficits [11,48,49]. One family of myelin associated diseases are the leukodystrophies, which refers to abnormal white matter. These diseases arise as a consequent of genetic defects that affect the generation or survival of oligodendrocytes and their associated myelin. Many of the leukodystrophies arise during development such as Pelizaeus-Merzbacher disease (PMD), which is a rare congenital disorder characterized by hypomyelination and is the result of duplication or missense mutation of the *PLP1* gene, eventually leading to abnormal or reduced myelin formation and oligodendroglial death [50]. Other disorders of hypomyelination that share similar characteristics with PMD have been characterized, and there is now close to 14 hereditary leukodystrophies reported in the literature [51]. A variation of the leukodystrophies is vanishing white matter disease. In this condition myelin appears to form and is then rapidly lost. The underlying mechanisms mediating vanishing white matter disease are not well established, although recent data suggests it is associated with changes in both oligodendrocytes and astrocytes [52,53]. Another congenital disorder of hypomyelination where the main pathology is in non-olidogendroglial cells is Alexander disease. This disease is caused by mutations in glial fibrillary acidic protein (GFAP) [54]. In general, because these diseases arise from genetic mutations directly or indirectly linked to myelin development and/or maintenance, their clinical sequelae are characteristically devastating with little or no treatment currently available.

**Figure 3:**
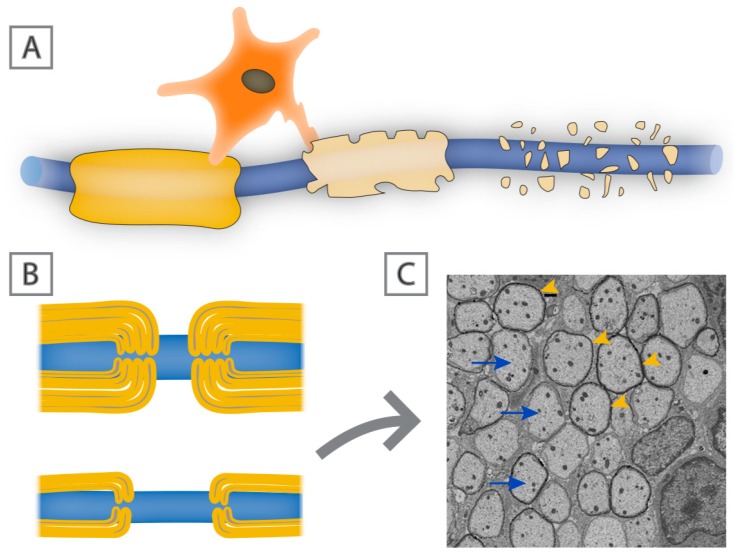
Demyelination vs. remyelination. Schematic of a myelinated axon (**A**), in which there is oligodendrocyte dysfunction and subsequent loss of myelin sheaths. Remyelinated axons ((**B**); lower panel) are characterized by thin myelin sheaths. (**C**) Electron micrograph of axons (arrows) in the dorsal white matter of the spinal cord in an adult rat showing thin myelin sheaths (arrowheads) following lysolecithin-induced demyelination.

The most common adult demyelinating disease of the CNS is multiple sclerosis [55]. MS can present in a number of different ways and may be a heterogeneous family of related conditions. The initial cause of MS is unknown. Genetic studies link the major histocampatablity complex (MHC), that reflects the role of the immune system in the pathology of the disease rather than its induction. Other risk factors include cigarette smoking, early exposure to viral infections such as EBV, low vitamin levels such as vitamin D, or loss of critical vitamin cofactors ([56,57,58], and refer to [59] for a comprehensive summary of the known risk factors associated with the development of MS). It seems as though multiple environmental cues may increase susceptibility to MS and many of these potential risk factors occur during early childhood, even though the disease generally does not manifest until adulthood. One possible explanation for this temporal separation is that during childhood and adolescence effective ongoing myelin repair masks the functional expression of demyelinating pathology. In its most common form, MS is considered to be an autoimmune condition that initially manifests itself as a relapsing-remitting disease with defined functional deficits from which individuals usually recover over time. It is understood that much of that initial recovery correlates with partial remyelination, which despite being refuted/debated for a long time, there is now strong histological and neuroimaging evidence to suggest there is constant remyelination in MS lesions [60,61,62,63]. However, the extent of remyelination seems to vary between people with MS, and even between lesion location in the same individual, which could in part be due to oligodendrocyte heterogeneity [5,64,65]. Following subsequent attacks, however, recovery is less complete and many individuals go on to develop chronic functional deficits secondary to progressive axonal loss. Interestingly, remyelination can usually be identified by the presence of invariably thin myelin sheaths (Figure 3B) [66]. Whether the progressive forms of the disease have a direct relationship to inefficient or incomplete remyelination is not clear, but there is evidence from histological studies implicating that migration of OPCs is affected in more chronic lesions, mainly owing to perturbed expression of extracellular matrix proteins, such as fibronectin and vitronectin [67,68,69]. Moreover, the differentiation of OPCs into myelinating oligodendrocytes is also impaired in chronic and progressive forms of MS [70,71]. The correlation of functional deficits with localized demyelination comes from imaging and postmortem analyses. A range of imaging modalities particularly MRI identify areas of white matter damage that can frequently be correlated with selective functional loss while pathological studies demonstrate areas of immune cell infiltration and astrocyte activation associated with demyelination. While areas of local demyelination are clearly a defining characteristic of MS, the precise degree of functional loss directly applicable to myelin loss rather than local inflammation or axonal damage is unresolved.

Relapses in the early stages of MS are associated with immune cell infiltration into the CNS, particularly T cells, and the symptoms can be partially alleviated through specific anti-inflammatory treatments [72]. A long-held assumption is that MS is an auto-immune CNS disease and that to treat it we need immunosuppressive or alternatively immunomodulatory therapy. There are currently 15 different MS therapies available all of which target some aspect of the immune system, either through blocking T cell entry into the CNS or by altering the overall immune repertoire [6]. An evolving theme in MS pathogenesis that has had a direct influence on the development of new therapeutics is the role of B cells, which was highlighted by the significant effect anti-CD20 therapies have had for people with MS, especially those with the primary progressive form of the disease [73,74,75]. There are no approved therapies to date, however, that address the failure of or inefficient remyelination in MS.

Another acquired demyelinating disease of the central nervous system is known as neuromyelitis optica (NMO), or Devic’s disease. Although NMO was initially thought to be a variant of MS, it is now recognized as a separate disease entity [76]. It preferentially affects the optic nerve (causing optic neuritis) and spinal cord (myelitis) with focal demyelination as its pathological hallmark. In a significant subset of people with NMO the pathology is associated with the generation of antibodies to aquaporin 4 and the presence of anti- aquaporin 4 antibodies is a definitive diagnostic test for NMO [77,78,79,80]. Aquaporin 4 is a water channel that is found selectively on the end feet of astrocytes abutting blood vessels. It seems likely the anti-aquaporin 4 antibodies target complement binding to astrocytes resulting in their death, disruption of the blood brain barrier, infiltration of immune cells and subsequent demyelination [81,82,83]. Currently available therapies for NMO are targeted at removal of circulating antibodies or complement activation rather than enhancement of remyelination.

In other CNS insults, myelin loss is not the primary target, rather oligodendrocyte death and myelin loss is a secondary consequence of the initial insult that contributes to further damage or dysfunction. For example, in the setting of ischemic stroke, neurons in the ischemic area are primarily affected, but their loss of viability leads to subsequent loss of myelin. Recent work suggests that this response is secondary to vesicular release of glutamate from axomyelinic structures, which in turn can interact directly with the myelin sheaths via NMDA receptors causing elevation of intracellular Ca^2+^ levels, and myelin decompaction and release [84,85]. A different mechanism by which ischemia causes elevation of intracellular Ca^2+^ and subsequent myelin damage is through elevation of extracellular K^+^, leading to an increase in intracellular H^+^ and activation of TRPA1 receptors [86]. Likewise, in spinal cord injury the initial insult results in direct damage to and severing of axons but secondary damage resulting from infiltration of immune cells and local inflammatory responses contribute to myelin and oligodendrocyte loss that again compromises the capacity of the tissue to recover/regenerate [87]. In both stroke and spinal cord injury, preventing oligodendrocyte and myelin loss has had a significant functional benefit for long term recovery [88]. Whether the mechanisms mediating myelin loss in these conditions are similar to those in diseases such as MS is not fully resolved. Given the heterogeneity of conditions with associated CNS demyelination, a broad range of different animal models have been developed to interrogate the pathological mechanisms and use that knowledge to develop remyelinating strategies.

## 6. Animal Models of Myelin Loss

A range of different animal models have been developed that are useful for enhancing our understanding of the mechanisms of demyelination and developing new therapeutic approaches to treat demyelinating diseases [89]. There are three general categories of animal models: those that depend on immune mediated demyelination; those that depend on chemical induced demyelination; and those that use genetic approaches to specifically ablate myelinating cells. Each of the models has strengths and weaknesses. For example, studies using the immune mediated models typified by experimental allergic encephalitis (EAE), reviewed in detail in Gold, et al. [90], include genetic elements of susceptibility and have allowed for the development of many of the current anti-inflammatory based treatments for MS that are currently on the market. In general, these treatments reduce inflammatory damage to the CNS and significantly enhance quality of life for people with MS, but do not effectively promote myelin repair (reviewed in detail in [6]). In a large, prospective study of 471 people with MS at one tertiary referral center, followed over a period of ten years, ultimate disease progression and significant disability was seen in 59% of people, regardless of how they were treated [91]. It is not surprising that therapies developed primarily using EAE models would fail to target myelin repair, since, in these animals, myelin destruction is continuous and simultaneous with any repair activity—either endogenous or therapeutically induced—making it difficult to assess the relative contribution of control of pathology and repair.

The second major category of demyelinating disease models are those involving the delivery of a gliotoxin that preferentially damages oligodendrocytes or other glia leading to demyelination. These models include local injection of lysolethicin or ethidium bromide or systemic delivery of cuprizone, and these models have the advantage that, once the toxin is removed, myelin undergoes spontaneous recovery [92,93,94]. This characteristic has allowed for the identification of therapeutic approaches that enhance myelin repair with little or no contribution from the immune system. The limitation of these models, however, is that they generate transient demyelination that undergoes spontaneous repair and as a result largely identify reagents that accelerate rather than initiate myelin repair.

The third general category of demyelinating models are those that generate demyelination through targeted ablation of oligodendrocytes. Several different paradigms have been developed to selectively eliminate oligodendrocytes including metabolic poisoning using cell type targeting of diphtheria toxin and the induction of cell type specific apoptosis through selective activation of the caspase cascade [95,96]. As yet, the use of such models has been restricted to mechanistic studies and not the development of new therapeutic approaches. Using combinations of the model systems discussed above, a number of myelin repair strategies have emerged. These include both cell-based and molecular strategies that alone or in combination with immunomodulators hold promise to alter the trajectory of CNS demyelinating conditions.

## 7. Therapies Promoting Remyelination

An extensive knowledge of mechanisms of demyelination over the past century, both from human and animal models has led to speculation on the key stages of remyelination. From developmental studies it seems likely that a key limiting step in myelin generation is probably going to be sufficient supply of OPCs from which proliferation, migration, and differentiation will take place allowing myelinating oligodendrocytes to start producing new myelin. This intricate and well-organized process has been the subject of many recent reviews [97,98,99]. It is no surprise therefore that one of the early regenerative approaches to treat demyelination revolved around re-stocking the tissues’ supply of precursor cells. Some of these experimental therapies are described below.

### 7.1. Oligodendrocyte Progenitor Cells

One potential approach to promoting remyelination in the CNS is to increase the number of OPCs that can develop into myelinating oligodendrocytes. OPCs have been derived from fetal and human brain tissue [100,101], human embryonic stem cells (hESCs) [102,103], and more recently from human induced pluripotent stem cells (hiPSCs) [104,105]. Advances in our understanding of the development of oligodendrocytes in both rodent and human systems resulted in the identification of cell type specific markers that allowed for the identification and isolation of relatively pure populations of OPCs. Building on rodent data, expression of CD140a PDGFRa identified through the binding of mAB A133 allowed unambiguous identification of human OPCs [106]. Isolation procedures and immunoselection processes from fetal tissue allowed for the generation of a cell population that could be expanded through selective growth factor addition and assayed for the ability to promote myelination following transplantation into selected animal models [100,101,107].

Following transplantation of human fetal or adult OPCs into immunocompromised myelin deficient rodent hosts, the OPCs were capable of differentiating and generating myelinating oligodendrocytes and astrocytes in the host brain [101,105,108]. These studies revealed a number of unexpected findings. First, the timing of integration of human cells was relatively slow compared to their rodent counterparts. Detection of human GFAP as a marker for astrocytes, or Myelin basic protein as a marker for myelinating oligodendrocytes revealed that the expansion of the cells took greater than 6 months. Second, although the host glia had populated the CNS prior to the expansion of the transplanted human cells, they were replaced over time by the human cells. Third, although the human cells were in a rodent environment they retained human cellular properties including a large size and distinct morphology. In other experiments, OPCs derived from hESCs integrated, migrated, and differentiated in the irradiated brain, producing cognitive and motor improvement [109]. More recently, hiPSC-derived oligodendrocytes were tested in vivo in MBP-deficient shiverer mice, and were shown to migrate and differentiate into myelinating cells. Together these studies demonstrated the capacity of cells isolated from the developing human CNS to generate a spectrum of OPCs in myelin repair. Translating these animal studies to the clinic is, however, challenging primarily due to the fact that the source of cells for transplantation is controversial. Fetal human neural cells are not readily obtained and have associated ethical issues, while OPCs derived from hESCs and/or hiPSCs are unproven in terms of safety, stability and reproducibility. Second, transplantation of non-host derived OPCs will require long term immunosuppression of the host to prevent rejection raising the potential for opportunistic infections. Finally, as with any replacement therapy it is unclear if the transplanted cells will suffer the same loss as endogenous host cells. Although these concerns are significant, the elegance of simply replacing lost oligodendrocytes to promote remyelination is a conceptually attractive strategy, in particular for hypomyelinating leukodystrophies where oligodendrocyte loss or dysfunction is the main pathological hallmark of the disease, and so replacement of defective oligodendrocytes would be more relevant. In MS, on the other hand, the presence of multi-focal lesions precludes the use of cell transplantation as an effective therapeutic strategy, since it would require the use of multiple injections.

### 7.2. MSCs in Myelin Repair

One stem cell population that has become increasingly attractive as a therapeutic for demyelinating diseases is mesenchymal stem cells (MSCs). Originally, MSCs were described due to their adhesive characteristics, the capacity to self-renew, and their ability to generate the major mesenchymal lineages including bone, fat, and cartilage. However, more recently it has been recognized that MSCs have profound immunomodulatory capacities and this has led to their development as a therapeutic for a number of conditions including demyelinating diseases of the nervous system [110,111,112,113]. Furthermore, and although the hypothesis that MSCs have the capacity to generate cells of the CNS is somewhat controversial and has been refuted [114], studies from a number of groups have demonstrated that in animal models of multiple sclerosis such as EAE, treatment with MSCs does at least in part promote oligodendrogenesis through the release of trophic factors, leading to enhanced functional remyelination [115,116,117,118]. Somewhat unexpectedly, MSCs derived from human bone marrow were equally effective in modifying disease as those from mice suggesting that the effector mechanisms are shared across species. Mechanistic analyses suggest that treatment with MSCs altered the balance between TH1 and TH2 cells leading to a reduction in proinflammatory cytokines such as TNFalpha and IL6, and an increase in anti-inflammatory cytokines such as IL10 [115,119,120]. In addition to their effects in modulating the immune system, MSCs have also been proposed to alter the specification of neural stem cells. In the presence of MSCs or their conditioned media, cultures of neural stem cells generate greater numbers of neurons and oligodendrocytes and reduced numbers of astrocytes consistent with their ability to promote recovery in the setting of disease [121,122]. These observations from multiple laboratories led to a number of phase I/II clinical trials in which autologous MSCs, mostly derived from bone marrow, were expanded in vitro and reinfused into individuals with relapsing-remitting or secondary progressive MS who have not responded to other disease modifying therapies [123,124]. In the majority of trials MSC infusion appeared relatively safe, however little or no benefit was observed as a result of MSC treatment in striking contrast to the benefits seen in mouse models [125,126,127]. Interestingly, an immediate immunomodulatory effect was reported in one trial, where an increase in CD4+CD25+ regulatory cells, and a reduction in the proportion of lymphocytes was seen [128]. A number of factors may explain the lack of MSC efficacy in human clinical trials including dosage, route of delivery, cell viability and cellular source. Evidence for evaluating the source of MSCs comes from studies suggesting that MSCs isolated from people with MS are far less effective at reducing disease burden and promoting repair than naïve cells when transplanted into animals with EAE [129]. Perhaps more compelling, bone marrow derived MSCs isolated from animals with EAE also show greatly reduced efficacy to reduce disease burden and promote repair when transplanted back into syngeneic EAE animals. Thus, the failure to see improvement in the MS clinical trials may reflect the use of autologous MSCs. Cataloging the molecular changes that occur in MSCs as a result of EAE reveal changes in more than 2000 genes mostly associated with immune responses and neural development [130]. Given the magnitude of these changes the future use of autologous MSCs for the treatment of MS is likely to be limited.

### 7.3. Bone Marrow Transplants

With advances in bone marrow stem cell ablation by chemotherapy and irradiation, several studies have adopted bone marrow transplantation as a potential treatment for MS. Initial outcomes from such approaches have been very encouraging with significant long-lasting reduction in disease burden and functional deterioration [131,132,133]. The exact mechanism underlying their benefit is not well understood. It seems likely, however, that the treatment results in a major resetting of the cellular repertoire of the immune system and with the deletion of CNS antigen-oriented T and B cells the pathology of the disease is reduced. Whether this reduction in pathology is sufficient to allow endogenous myelin repair in the CNS or it simply prevents further insults has yet to be determined. There are, however, a number of concerns with bone marrow transplant approaches. First, the stem cell depletion regime is very harsh and carries a significant risk particularly for individuals already severely compromised by disease; in one multi-center cohort study of 281 individuals, transplant related mortality was 2.8% within 100 days, and the overall survival at 5 years was 93% [131]. Second, it is unclear whether over time CNS antigen directed T and B cells will reemerge resulting in a recurrence of the disease. Third, the treatment is currently expensive and presumably negates prior immune protection leaving recipients potentially susceptible to opportunistic infections. While bone marrow transplantation may be an attractive option for some individuals with autoimmune diseases, more targeted molecular therapies that promote myelin repair in the CNS are clearly needed.

### 7.4. Molecular Mediators of Myelin Repair

In many conditions, both in animal models and in humans, remyelination is quite effective and it seems likely that demyelinating diseases largely become symptomatic when the pathologic disease activity exceeds the capability of the tissue for repair. Such an imbalance may occur if the disease is overwhelmingly severe, or the repair process is compromised. Analysis of post mortem tissue suggests that in many cases of MS the process of myelin repair is compromised, and this has led to concerted efforts to enhance remyelination through either modification of the lesion environment or enhancing the capacity of OPCs to generate myelin in the adult animal. Currently, evidence supports the notion that one of the rate limiting steps in myelin repair is the differentiation of OPCs into myelinating oligodendrocytes. For example, distinct subpopulations of MS lesions contain demyelinated axons adjacent to cells that have the characteristics of OPCs but have failed to differentiate and myelinate nearby axons [20,67,134]. In the absence of myelination such axons are more vulnerable to damage and loss of axons ultimately leads to irreversible functional loss.

Two general strategies can promote remyelination by oligodendrocytes. The first involves the neutralization of inhibitory signals that block the formation of myelin by oligodendrocytes. Several such signals have been identified including the expression of high levels of poly sialic acid on the surface of axons and the expression of LINGO1 on OPCs and possibly axons. LINGO1 is a leucine repeat rich cell surface molecule, initially identified in axons as part of the Nogo receptor complex and has an inhibitory role in axon regeneration [135]. Its expression in oligodendrocytes is also associated with negative regulatory effects, by way of limiting oligodendroglial differentiation and myelination [135,136]. Inhibition of LINGO1 with specific antibodies promotes oligodendrocyte differentiation, enhances myelination and reduces functional deficits in EAE [136,137]. Initial clinical trials with anti-LINGO1 (opicinumab) have not been as successful as the animal models had predicted [138,139]. Specifically, no difference was observed in remyelination as measured by full-field visual evoked potentials (VEP) at 24 weeks. However, further refinement of patient selection and treatment paradigms will likely provide increased benefit, and a new trial is underway to assess the efficacy of opicinumab as an add-on therapy in relapsing remitting MS [140]. Another potential target is the muscarinic acetylcholine receptors (mAChRs); their activation in cultured OPCs resulted in lower expression of myelin protein in mature oligodendrocytes, and increased expression of PDGFRa, both of which act to inhibit oligodendrocyte differentiation [141]. Consistent with this finding, benztropine, an inhibitor of the muscarinic receptor, was identified in a high throughput screen utilizing purified rat OPCs, and subsequently found to enhance remyelination and functional recovery in EAE and cuprizone-induced demyelination models [142]. With the realization that oligodendrocytes are capable of myelinating inert fibers of the correct size, an inert micro pillar approach also identified another anti-muscarinic agent, clemastine, as a promoter of myelination [143]. Recent clinical trial data suggest it may have some efficacy in MS, by showing shorter VEP latencies in the treatment group, a measure of optic nerve remyelination [144]. Other less well developed signaling pathways with a negative regulatory role on oligodendrocyte maturation and myelination, such as the Wnt pathway, are still being studied, and targeted therapeutic options are currently under development [145,146].

The second general approach deals with enhancement of pro-differentiation signals. With the emergence of more highly developed stem cell technologies, a number of positive regulators of oligodendrocyte development have been identified. For example, the ability to generate large numbers of OPCs using epiblast derived or IPS cell technology has facilitated effective high through-put screening that led to the identification of miconazole—an anti-fungal, and clobetasol—a glucocorticoid receptor agonist, as promoters of oligodendrocyte differentiation [147]. In all cases, rodent remyelinating systems have demonstrated that factors promoting oligodendrocyte differentiation also enhance the rate of myelin repair in vivo.

Other therapies that have been studied recently address different aspects of oligodendrocyte physiology in an effort to indirectly promote remyelination. For example, biotin is a cofactor for essential carboxylases, one of which (acetyl-CoA carboxylase) is expressed in oligodendrocytes. It was recently shown in people with progressive MS that high dose biotin has had a favorable effect on functional disability [148]. It is still unclear whether the functional improvement was secondary to myelin repair, which is thought to be secondary to fatty acid synthesis, or to axonal protection from enhanced energy production.

## 8. Challenges to Developing Remyelinating Therapies

Efforts to identify remyelinating therapies in the CNS are still in their infancy. Compared to the effort that has been committed to developing modulators of the immune system, relatively little work has been focused on remyelination. Even with increased efforts, however, the barriers to success are formidable. For example, much of the current work is focused on enhancing oligodendrocyte differentiation, and yet the evidence that this is the primary rate limiting step in remyelination failure in MS and other demyelinating diseases is circumstantial. Indeed, the agents currently identified in a variety of screening studies appear to accelerate remyelination, but it is unclear whether they are capable of initiating repair. This uncertainty stems in part from the lack of appropriate rodent models for chronic demyelination. While EAE has been extremely useful for relapsing-remitting MS, there is currently a lack of models of progressive, chronic MS.

An additional hurdle to developing effective repair strategies is the complexity of the pathology. In addition to axons, cells of the oligodendrocyte lineage, and immune cells, multiple other cell types are involved in both pathology and likely repair. These include astrocytes, microglia, pericytes, neural stem cells, and cells of the vasculature. Developing therapies based on a single cell type response misses the intrinsic complexity of the lesion that may dictate outcomes. Finally, issues of species specificity and perhaps more importantly scale are critical for effective translation into the clinic. While small lesions may repair effectively, once they attain a critical size repair may fail. Generating models of large lesion size likely requires animal models other than rodents.

## 9. Conclusions

In recent years great progress has been made in developing new therapeutic approaches for the treatment of demyelinating diseases. While there is a large number of different approaches to the treatment of demyelinating diseases such as MS, the majority of these approaches are focused on reducing the impact of the immune system in promoting the pathogenesis. However, none of the currently available therapies is able to provide a cure or effectively terminate the pathology, as evidenced by the propensity for recurrence of myelin loss and eventual functional decline. There is a dire need for more targeted therapeutics that can restore myelin and protect demyelinated axonal processes. One of the key advances of the last decade has to be our expanding knowledge of some of the developmental mechanisms governing myelination, but more importantly also spontaneous remyelination. We are unraveling a great deal of mystery pertaining to a once un-treatable demyelinating disease, MS, in humans with the hope that this will rapidly translate into new effective therapies.
